# Real-world study of direct medical and indirect costs and time spent in healthcare in patients with chronic graft versus host disease

**DOI:** 10.1007/s10198-020-01249-x

**Published:** 2020-12-04

**Authors:** Frida Schain, Nurgul Batyrbekova, Johan Liwing, Simona Baculea, Thomas Webb, Mats Remberger, Jonas Mattsson

**Affiliations:** 1 Janssen Global Services, Stockholm, Sweden; 2 Department of Medicine, Division of Hematology, Karolinska Institutet, Solna, Sweden; 3 Schain Research, Bromma, Sweden; 4 Department of Medical Epidemiology and Biostatistics, Karolinska Institutet, Stockholm, Sweden; 5 Scandinavian Development Services, Stockholm, Sweden; 6 Department of Medicine, Division of Hematology, Karolinska Institutet, Huddinge, Sweden; 7 Janssen Global Services, High Wycombe, UK; 8 KFUE, Uppsala University Hospital and Institution of Medical Science, Uppsala University, Uppsala, Sweden; 9 Department of Oncology and Pathology, Karolinska Institutet, Stockholm, Sweden; 10 Gloria and Seymour Epstein Chair in Cell Therapy and Transplantation, Princess Margaret Cancer Centre, University of Toronto, Toronto, ON Canada; 11 Department of Medicine, University of Toronto, Toronto, Canada

**Keywords:** Chronic graft versus host disease, Economic burden, Direct medical costs, Indirect costs, Sweden, E24, H51, I18

## Abstract

Chronic graft versus host disease (cGVHD) is a debilitating and costly complication following haemopoietic stem cell transplantation (HSCT). This study describes the economic burden associated with cGVHD. Direct costs associated with specialised healthcare utilisation (inpatient admissions and outpatient visits), as well as indirect costs associated with sickness absence-associated productivity loss were estimated in patients who underwent allogeneic HSCT in Sweden between 2006 and 2015, linking population-based health and economic registers. To capture the period of chronic GVHD, patients were included who survived > 182 days post-HSCT (start of follow-up), and cGVHD was classified based on patient treatment records to correct for any diagnosis underreporting. Patients were classified as ‘non-cGVHD’ if they received no immunosuppressive treatment, ‘mild cGVHD’ if they received only systemic corticosteroid treatment or immunosuppressive treatment, or ‘moderate–severe cGVHD’ if they received extracorporeal photopheresis (ECP) only, corticosteroid treatment and immunosuppressive treatment, or systemic corticosteroid treatment and ECP treatments. Patients with moderate–severe cGVHD spent more time in healthcare, had higher healthcare resource costs and higher sickness absence-related productivity loss compared to patients with non- or mild cGVHD. The cumulative total costs during the first 3 years of follow-up were EUR 14,887,599, EUR 20,544,056, and EUR 47,811,835 for non-, mild, and moderate–severe groups, respectively. The long-term costs incurred with cGVHD following HSCT continue to be very high and significantly impacted by cGVHD severity. This study adds real-world health resource and economic insight relevant for policy-makers and healthcare providers when considering the clinical challenge of balancing immunosuppression to reduce cGVHD.

**Electronic supplementary material:**

The online version of this article (10.1007/s10198-020-01249-x) contains supplementary material, which is available to authorized users.

## Introduction

In 2012, there were 31,926 reported allogeneic haemopoietic stem cell transplantations (HSCTs) in 77 of the 79 countries known to have performed HSCTs [1]. Chronic graft versus host disease (cGVHD) is a debilitating long-term complication associated with HSCT [2], affecting approximately 30%–70% of HSCT recipients [3–5], with many associated symptoms presenting in the first year post-HSCT [6]. An incidence model estimated that the number of patients with mild, moderate, and severe cGVHD in Europe will rise from 908 to 977, 1375 to 1481, and 935 to 1007, respectively, between 2019 and 2023 [7]. The skin, eyes, lungs, mouth, gastrointestinal tract, musculoskeletal system, genitals, liver, and immune system are commonly affected [8, 9]. cGVHD is associated with a lower risk of relapse of haematological malignancy [10, 11]. However, it is also associated with an increased risk for non-relapse-related mortality and inferior overall survival in patients with more severe disease [10, 12, 13]. Further, cGVHD adversely affects patients’ health-related quality of life (QoL) and functional status, with disease severity and organ involvement being key risk factors for such outcomes [14–16].

The disease has been recently well characterized by the 2005/2014 National Institutes of Health (NIH) Consensus Conference that standardized the criteria for the diagnosis of cGVHD [17]. These criteria are the standard for implementing decisions on cGVHD treatment and enrolment in clinical trials. Data from an international survey to establish the uptake of NIH recommendations suggest, however, that these recommendations were used only by 54%–69% of respondents (including physicians, nurse practitioners, and physicians’ assistants) [18]. Without widespread adoption of international standards, there is a potential risk of misdiagnosing or under-diagnosing cGVHD. Through Swedish national registers, we conducted a retrospective, population-based, longitudinal study of patients with cGVHD [19]. Instead of a method based on presentation, such as the NIH criteria, we have established a method that considered the timing and extent to which patients received systemic immunosuppressive cGVHD treatment following HSCT (Supplementary Fig. 1 and 2 in Online Resource; [19]). We found a significantly higher incidence of cGVHD (71.9%) in long-term survivors (> 182 days post-HSCT) than previously reported in Sweden (38.0%) [20], and significantly higher morbidity rates for patients with moderate–severe cGVHD versus those with non- and mild cGVHD [19].

Studies have shown that cGVHD remains a barrier to reducing the cost burden for long-term survivors after HSCT. Although a number of studies have assessed this cost burden, there are few recent studies of the burden of cGVHD following HSCT [21–24]. Notably, an economic study in France (*n* = 134) in adults with acute leukaemia showed that patients with cGVHD required an additional 12 days of hospitalization, at a cost of EUR 765 per day [22]. In a recent pharmacoeconomic analysis in Tunisia of patients who had undergone HSCT with acute and chronic leukaemia, the average direct cost of managing cGVHD was threefold higher than for those without cGVHD [USD 19,523 (*n* = 13) versus 6073 (*n* = 19), respectively; *p* = 0.032] [24]. A recent US claims study (*n* = 523) reported steroid-resistant cGVHD patients spent significantly more days in healthcare and had higher healthcare resource utilisation costs, particularly inpatient costs, compared to non-cGVHD patients in the first 2 years post-HSCT [25]. In Belgium, cost of inpatient care alone for cGVHD following HSCT was estimated at approximately EUR 2600 per month [26].

Beyond direct health care cost, indirect costs also have a significant contribution to the total cost of illness [27]. Studies published since 2010 have reported that cGVHD burden of illness translates into a higher cost to society and loss of productivity from those patients with cGVHD who are unable to work [16, 28, 29]. A cost-of-illness analysis in the United States estimated that the financial burden of cGVHD in patients (*n* = 44,450) would accumulate a total of 605,631 years in lost wages, and a loss of productivity; only 37.5% of patients with cGVHD would return to work [28]. Similarly, in a longitudinal study that evaluated the QoL and number returning to work, 41.0% of patients with cGVHD returned to work compared with 95.0% without cGVHD [16]. Further, a recent questionnaire study (*n* = 383) by the North American cGVHD Consortium (Canada and United States) assessed the insurance, employment, and financial challenges endured by patients with cGVHD who had undergone HSCT. The study showed that, of respondents who reported a financial burden, 40.0% included an inability to return to work as a cause [29]. While studies have examined the considerable economic cost of cGVHD and have shown the association of HSCT with reduced work capacity, there are insufficient data concerning productivity and sickness absence, and resulting economic costs in patients incurred by treating cGVHD following haematological malignancies. Additionally, there is a lack of European data on indirect costs, with the majority of data from North America [16, 28, 29]. Considering direct healthcare costs in parallel with the indirect cost [27] is important to provide a more integrated insight into the economic burden of cGVHD.

More research is needed to better understand the costs of cGVHD, relevant to both patients and to healthcare systems. To address this unmet need, we conducted a population-based longitudinal study of patients with cGVHD through Swedish national registers from 2006 to 2017. Swedish national registers were used as they contain comprehensive details of treatment, along with inpatient and outpatient visits [30]. This is the first real-world health economic study in cGVHD that uses a model to correct for clinical underdiagnosis [19], thereby contributing a more comprehensive analysis of economic outcomes in the recent management of cGVHD.

## Methodology

### Study population identification and characteristics

Instead of the standard 2014 NIH criteria based on the degree of organ impact and functional impairment [17], we used a method that considered the timing and extent to which patients received cGVHD treatment to classify those who had developed cGVHD [19] (Supplementary Fig. 1 and 2 in Online Resource). NIH criteria advise systemic or local treatment for mild cGVHD [17], whereas our classification defines mild cGVHD based on the extent of systemic treatment [19]. We searched for the treatments that are recommended by these guidelines and those that correspond to cGVHD severity. Patients treated with topical corticosteroids, with or without the prescription of a doctor, did not meet our classification of cGVHD. Further, our classification may not capture all patients with mild disease, specifically those with mild cGVHD limited to a single organ [17].

After ethics committee approval (dnr 2017/1716-31/1), we used nationwide Swedish population-based registers, which are managed by the National Board of Health and Welfare and Statistics Sweden (SCB). Patients who underwent HSCT were identified in the Patient Register. These patients were linked to the Cancer Register, the Prescribed Drug Register, the Cause of Death Register, and the Longitudinal Integration Database for Health Insurance and Labour Market Studies (LISA) Register. The Prescribed Drug Register was established in 2006, which provided the start date of our study period. We identified 2147 patients who underwent HSCT from 2006 to 2015. Patients were excluded if they had duplicated proxy identification numbers, were aged < 18 or > 75 years, had no record of haematological malignancy prior to HSCT, and survived < 182 days following HSCT.

Patients were included who survived ≥ 182 days post-HSCT, a time point based on the common presentation time of cGVHD [4]. Using criteria developed by investigators, patients classified as having cGVHD were defined as having mild or moderate-severe cGVHD, based on timing and extent of treatments commonly used for cGVHD (Supplementary Fig 1 and 2 in Online Resource). Patients who did not receive systemic immunosuppressive treatment were classified as non-cGVHD. Patients with mild cGVHD were defined as those who received only systemic corticosteroid treatment for > 3 months; whose last date of systemic corticosteroid treatment ended < 3 months before censoring; whose last date of corticosteroid treatment ended < 6 months (180 days) before death; who received immunosuppressive treatment only. Patients with moderate–severe cGVHD were defined as those who received ECP only, or corticosteroid treatment and immunosuppressive treatment, or systemic corticosteroid treatment and ECP. For the cGVHD classifications, we defined 3 months as 90 days and 6 months as 180 days.

We included 1246 patients who underwent HSCT from 2006 to 2015 and met our inclusion criteria. Of these, 28.1% were classified as non-cGVHD and 71.9% as cGVHD (27.7% as mild cGVHD and 44.2% as moderate–severe cGVHD) (Table 1). Age, sex, calendar year, source for HSCT, salary, and education level at the start of follow-up (182 days post-HSCT) were comparable among the three groups (Table 1). There was, however, heterogeneity among the three groups for those using matched related or unrelated donors *(p* < 0.0001) (Table 1).

**Table 1:** Demographic characteristics per group at the start of follow-up time (182 days post-HSCT)

	Non-cGVHD	Mild cGVHD	Moderate-severe cGVHD	*p* value	Overall
Total, *n* (%)	350 (28.1)	345 (27.7)	551 (44.2)		1246 (100)
Sex, *n* (%)					
Men	206 (59)	192 (56)	323 (59)	0.618	721 (58)
Women	144 (41)	153 (44)	228 (41)		525 (42)
Median age, years (IQR 1–3)	53 (41–61)	52 (40–61)	52 (39–62)	0.985	52 (40–61)
Calendar year, *n* (%)					
2006–2010	116 (33)	122 (35)	195 (35)	0.758	433 (35)
2011–2015	234 (67)	223 (65)	356 (65)		813 (65)
Donor, *n* (%)					
Related	95 (27)	88 (26)	209 (38)	< 0.0001	392 (31)
Unrelated	255 (73)	257 (74)	342 (62)		854 (69)
Education level, *n* (%)					
Below university	222 (63)	220 (64)	345 (63)	0.994	787 (63)
University and higher	126 (36)	123 (36)	202 (37)		451 (36)
Missing	2 (1)	2 (1)	4 (1)		8 (1)
Salary (EUR)^a^ (*n* = 1095)					
0–9650	72 (24)	79 (26)	136 (28)	0.47	287 (26)
9752–29,158	105 (35)	123 (40)	173 (35)		401 (37)
29,256–38,910	74 (25)	65 (21)	106 (22)		245 (22)
39,008	47 (16)	37 (12)	78 (16)		162 (15)

*cGVHD* chronic graft versus host disease, *HSCT* allogeneic haematopoietic stem cell transplantation, *IQR* interquartile range

^a^Only includes patients ≤ 65 years

## Analyses

### Statistical analysis

For statistical analyses, the end of the 182-day period post-HSCT was the index date (start of observation time) (Supplementary Fig. 1. in Online Resource). Univariate analysis (Chi-square and Kruskal–Wallis) was used to compare the demographic and baseline differences among the three cGVHD groups (patients with non-, mild, and moderate-severe cGVHD). Our analysis does not take into account the costs for HSCT or those incurred in the first 182 days post-HSCT.

### Salary estimate

Patients’ salaries at 1–10 years from the start of observation time were estimated using the calendar year–specific total cash gross salary from employment, active business activities, and work-related compensation from the LISA register [31]. We accounted for payroll taxes and social fees by multiplying the annual salary by 1.3142 [32].

### Healthcare resource utilisation and direct medical costs

Average time spent (days) in healthcare was estimated using records in the Patient Register for outpatient visits and inpatient admissions per follow-up year. Direct medical costs associated with specialised healthcare (inpatient and outpatient) were estimated using the diagnosis-related group (DRG) codes provided in the Patient Register for each healthcare visit [33]. DRG stratifies patients of similar resource use based on diagnosis, procedures performed, age, sex and status at discharge [33]. DRG codes were converted to costs using DRG weights published annually by the Swedish Board of Health and Welfare to inform a total generalised complete care cost (including clinical care, diagnostics and hospital medicine costs). Primary care visits and pharmacy medications are not included. Incidence rate ratios (IRRs) were derived through multivariate negative binomial models to compare health resource utilisation between age groups and sexes. IRRs were adjusted for sex and age at the start of observation time and follow-up year. The percentile bootstrap method was used for computing 95% confidence intervals (CIs).

### Sickness absence and indirect cost

Indirect cost describes losses in productivity due to absence from work using the human capital approach [34]. This approach uses salary (including social taxes) as a proxy for the value of productivity, cumulatively reflecting the lost value of production impacting society due to reduced labor force. The indirect cost dataset comprised 1095 patients, as patients aged > 65 years (the typical retirement age in Sweden) during follow-up (*n* = 148) and those with missing salary data (*n* = 3) were excluded from this productivity analysis. Sickness absence rates were derived from the LISA Register. We defined sickness absence days as the total number of days during the specific period that a patient was absent from work due to sickness (+ 14 days that are employer paid and therefore not captured in the population register), and/or the number of days a patient received sickness compensation, sickness benefits, activity allowance, or early-retirement pension due to sickness. A year is defined as 365.25 days and a month as 30.4 days.

The sickness absence rate for the follow-up year was defined as the total number of sickness absence days divided by the total contributed follow-up time. Further description is provided in the “Methods” of the Online Resource. Indirect costs for each follow-up year were calculated as the number of sickness absence days in this year and multiplied by the average daily salary for the given follow-up year. The IRRs from the multivariate negative binomial regression with 95% CI from the percentile bootstrap method were used to compare sickness absence rates among the three cGVHD groups, while adjusting for sex and age at the start of observation time and follow-up year.

### Total costs

Total costs per follow-up year were calculated as a sum of direct medical and indirect productivity costs.

### Inflation and currency adjustment

We collected all costs in SEK, adjusted for inflation for 2018, and converted these costs to EUR using the 2018 average conversion rate SEK 1 = EUR 0.09752 [35].

## Results

### Direct medical costs

**Fig. 1:**
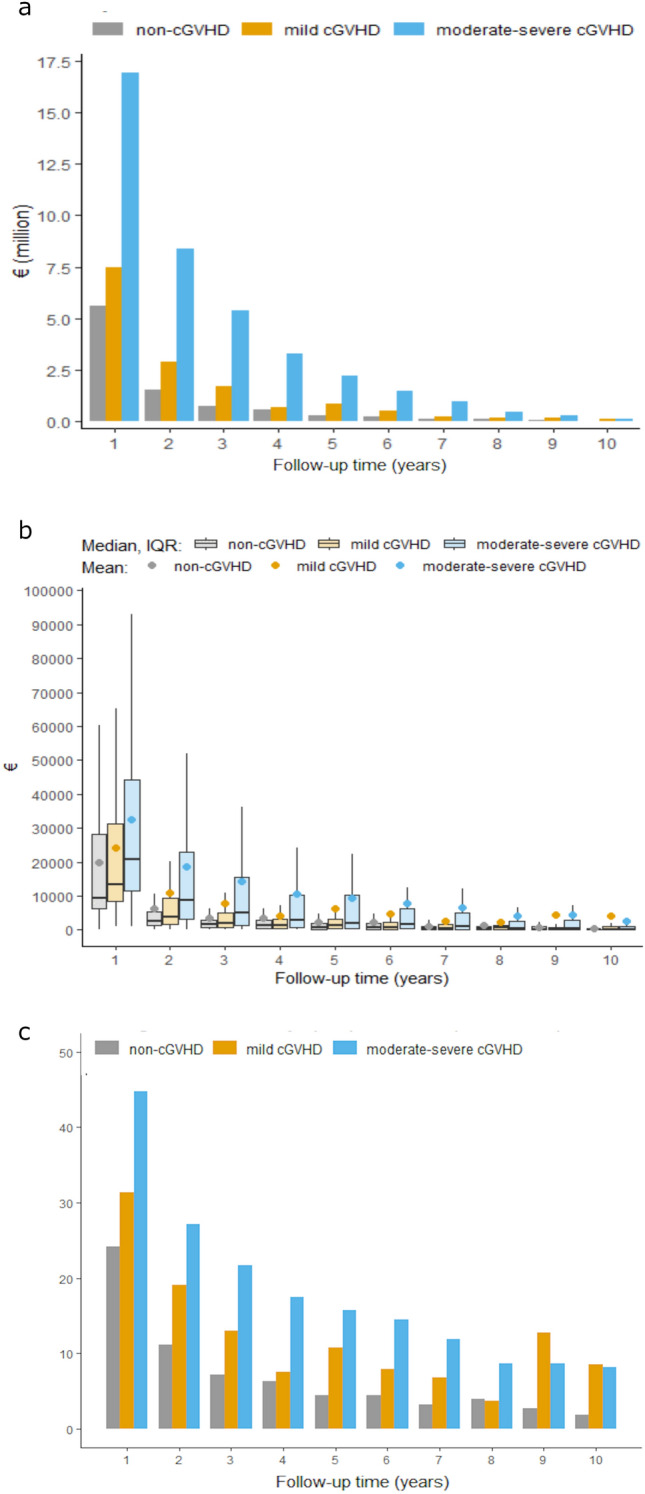
Direct medical costs and days spent in healthcare for patients with non-, mild and moderate–severe cGVHD, per follow-up year from 182 days post-HSCT. Cumulative direct medical all patient costs (**a**) and per patient cost (**b**) by cGVHD severity. Number of days spent in healthcare (inpatient and outpatient) (**c**). *cGVHD* chronic graft versus host disease, *IQR* interquartile range

To get an overall view of direct medical costs associated with specialised healthcare, we assessed the costs incurred in inpatient and outpatient care during 1–10 years from the observation time (Fig. 1; Supplementary Table 1 in Online Resource). Over the 10-year period, the first year of follow-up was the most costly and there was a decrease in both cumulative (Fig. 1a) and per person costs (Fig. 1b) at each subsequent follow-up year in each patient group (non-cGVHD, mild cGVHD, and moderate–severe cGVHD). Generally, patients with moderate–severe cGVHD showed the greatest costs throughout the follow-up period (Fig. 1a, b; Supplementary Table 1 in Online Resource).

The cumulative direct costs (in- and outpatient care), borne by the Swedish universal healthcare system, of all patients in the first three years of follow-up were estimated at EUR 7,845,114, EUR 11,971,307, and EUR 30,643,472 for patients with non-, mild, and moderate–severe cGVHD, respectively (Fig. 1a; Supplementary Table 1 in Online Resources). Costs per person-year were also higher for moderate-severe cGVHD patients across subsequent follow-up years (Fig. 1b). The cumulative average per person cost for the first 3 years of follow-up was EUR 29,812, EUR 43,025 and EUR 65,559 for patients with non-, mild, and moderate–severe cGVHD, respectively (Fig. 1b; Supplementary Table 1 in Online Resources).

Inpatient care costs were generally higher than outpatient care costs in patients with moderate-severe cGVHD (Supplementary Table 1 in Online Resources). At 1–3, 6, and 8–10 years of follow-up, patients with mild cGVHD also showed higher inpatient than outpatient costs. This finding was not seen in patients with non-cGVHD, where outpatient costs were higher at each follow-up year. It should be noted that the number of patients was low, and these numbers declined over time.

Moderate–severe cGVHD patients spent more time in healthcare than non or mild cGVHD patients. In the first year, moderate–severe patients spent on average 44.7 days in healthcare (inpatient and outpatient) per patient, compared to 24.2 and 31.3 days for non- and mild cGVHD patients (Fig. 1c). While this dropped to an average 27.2 days in the second follow-up year (non cGVHD 11.1 days; mild cGVHD 19.0 days), moderate–severe cGVHD patients continued to generally spend a higher number of days in healthcare across all follow-up years. We have shown previously that higher outpatient resource usage was prominent in moderate–severe cGVHD patients [19]. Multivariate analysis of overall time in care showed that, compared with patients aged 18–39 years, patients aged 40–59 years utilised more healthcare resources (IRR [95% CI] 1.18 [1.02–1.39]), but this trend was not seen in those aged 60–75 years (IRR [95% CI] 1.14 [0.94–1.36]). Compared with men, there was no difference in time spent in healthcare among women (IRR [95% CI] 1.02 [0.90–1.17]).

### Indirect costs

**Fig. 2:**
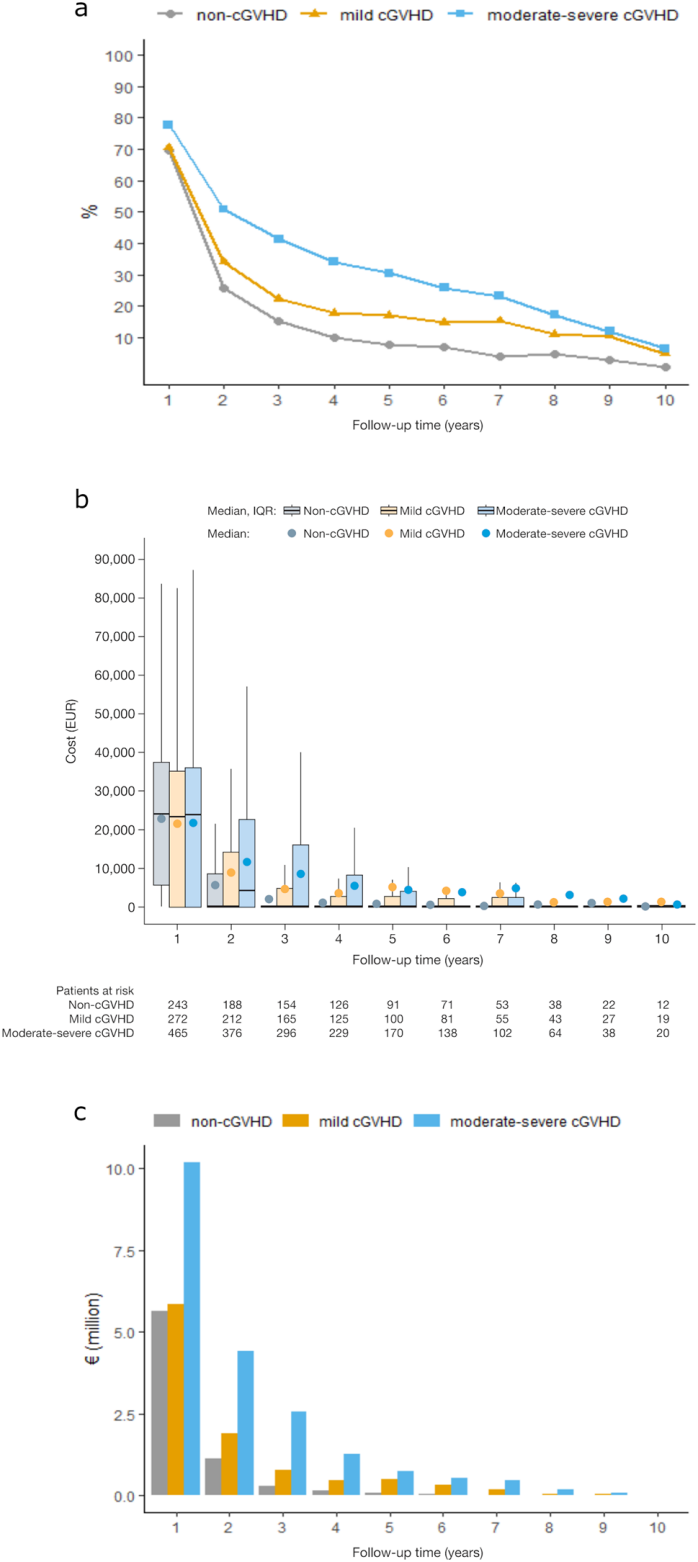
Indirect costs due to sickness absence-associated productivity loss in patients with non-, mild and moderate–severe cGVHD, per follow-up year from 182 days post-HSCT. Sickness absence rates (**a**), cumulative all patient (**b**) per patient productivity loss costs by cGVHD severity. *cGVHD* chronic graft versus host disease, *IQR* interquartile range

To determine indirect costs associated with lost productivity, we assessed these costs at 1–10 years from the 182 post-HSCT observation start time (Fig. 2; Supplementary Table 2 in Online Resources). Overall, the highest indirect costs were seen in patients with moderate-severe cGVHD.

During the first year (*n* = 1095) from the start of follow-up, we found that the sickness absence rate for patients with moderate-severe cGVHD was 77.7% versus 69.7% for patients with non-cGVHD and 70.5% for patients with mild cGVHD (Fig. 2a). Showing a similar trend, during the third year (*n* = 635), the sickness absence rate for patients with moderate–severe cGVHD was 41.4% versus 15.1% for patients with non-cGVHD and 22.4% for patients with mild cGVHD (Fig. 2a). Multivariate analysis revealed that the incidence of sickness absence increased with increasing cGVHD severity, and that during the overall study period (*n* = 1095), sickness absence rate ratios were significantly lower for patients with non-cGVHD (IRR [95% CI] 0.59 [0.53–0.65]) and mild cGVHD (IRR [95% CI] 0.72 [0.66–0.79]) cGVHD compared with moderate-severe cGVHD.

Cumulative indirect cost for all patients was highest for the moderate–severe cGVHD patient group in the first year of follow-up, and this trend remained for each subsequent follow-up year (Fig. 2b; Supplementary Table 2 in Online Resource). We found that the mean indirect costs of all patients in the first 3 years of follow-up were EUR 7,042,484, EUR 8,572,749, and EUR 17,168,364 for patients with non-, mild, and moderate–severe cGVHD, respectively.

The mean cost per patient was similar among cGVHD groups during the first follow-up year (Fig. 2c; Supplementary Table 2 in Online Resource). After the first year, while indirect costs decreased for all cGVHD groups, it remained persistently higher for moderate-severe patients compared to non- or mild cGVHD patients. Mean indirect costs per patient increased with increasing cGVHD severity in all subsequent years from the second follow-up year, except for the tenth year (Fig. 2c; Supplementary Table 2 in Online Resource).

### Total cost

**Fig. 3:**
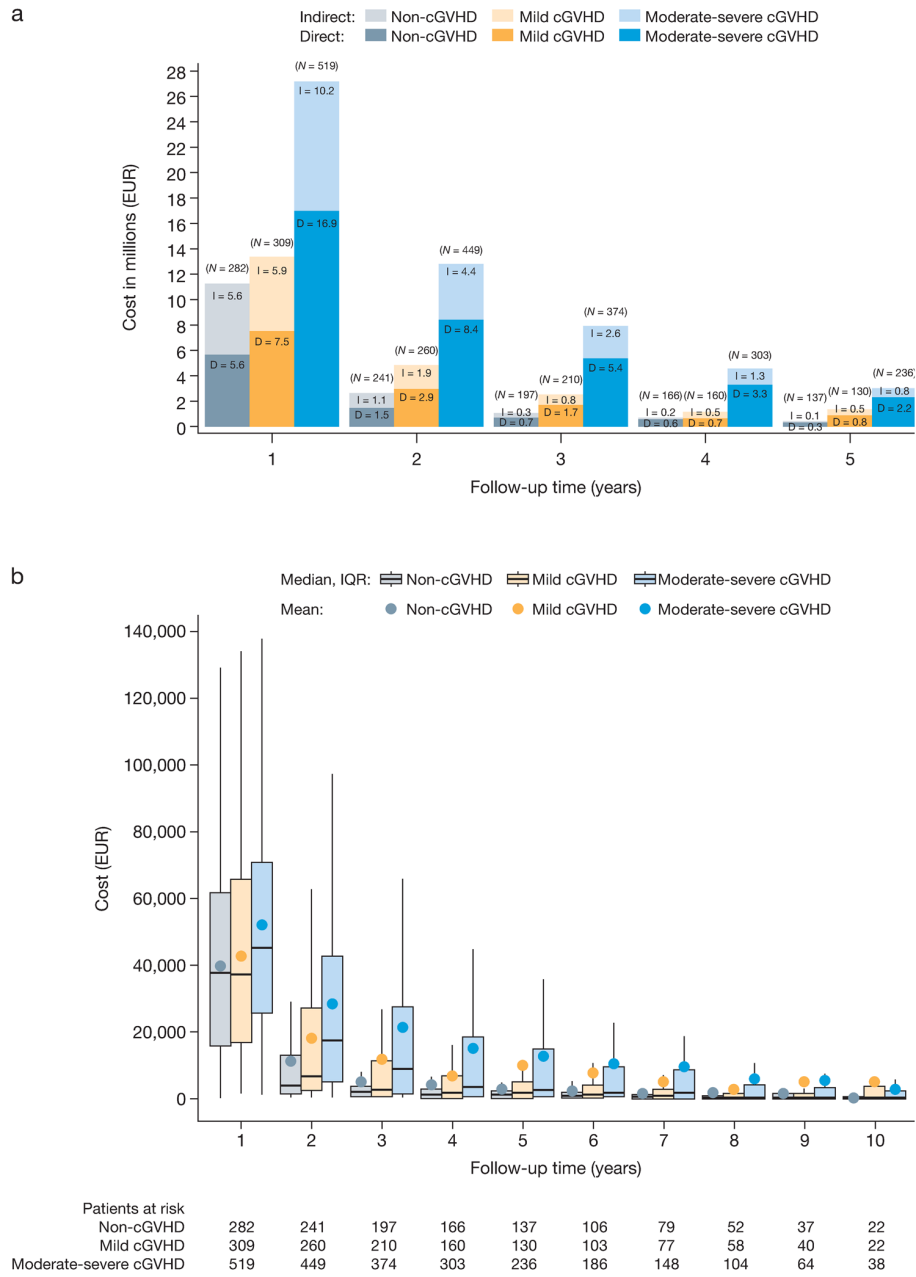
Total costs combining direct medical and indirect productivity costs in patients with non-, mild and moderate–severe cGVHD, per follow-up year from 182 days post-HSCT. Cumulative all patient total cost, with proportion of direct medical cost (dark shading) and indirect productivity cost (light shading) (**a**). Total costs per patient per year (**b**). *cGVHD* chronic graft versus host disease, *IQR* interquartile range*, D* direct medical cost, *I* indirect medical cost

Finally, we assessed total cost; defined as combined direct medical (inpatient and outpatient) and indirect costs. Generally, the mean total (Fig. 3a) and per-person (Fig. 3b) total costs decreased across follow-up years in each patient group over the 10-year period. However, the greatest total costs were shown in patients with moderate-severe cGVHD. Of total costs, direct costs were the largest contributor to total costs (Fig. 3a). The total cumulative all patient cost for first three years of follow-up from our start date was EUR 14,887,599, EUR 20,544,056, and EUR 47,811,835 for patients with non-, mild, and moderate-severe cGVHD, respectively (Fig. 3a; Supplementary Table 3 in Online Resources).

The mean total cost per patient during the first follow-up year was highest for with moderate-severe cGVHD compared to patients with non- and mild cGVHD. This finding was seen in all subsequent follow-up years except for the tenth year (Fig. 3b; Supplementary Table 3 in Online Resource). The cumulative total cost per patient over the first 3 years of follow-up was EUR 55,859, EUR 73,000 and EUR 101,777 (Fig. 3b; Supplementary Table 3 in Online Resources).

## Discussion

In this study, we aimed to estimate healthcare costs, time spent in healthcare, sickness absence, and productivity loss of patients with cGVHD in Sweden who survived 182 days post-HSCT. Despite the debilitating and long-term impact of cGVHD in a large proportion of patients who undergo HSCT, there is a paucity of recent studies addressing the economic costs variables in the management of cGVHD, and even fewer from a European perspective. Using Swedish population-wide patient health and economic registers, we provide a comprehensive analysis of direct healthcare cost and indirect costs due to productivity loss. First, using our novel real-world treatment-based criteria [19], we more accurately identified patients treated for cGVHD; a diagnosis shown to be underreported due to low adoption of standardized criteria. This means we are able to present a more complete picture of the total societal costs in the management of cGVHD. Second, Sweden is renowned as a world leader in comprehensive patient registers [36], and data from a centralized health and social security means data is derived from the whole population without selection bias. Third, unique personal identification numbers facilitate patient-level linkage of health and economic registers, and we use individual patient salary to more accurately describe productivity loss, rather than population-based averages often used in such analyses.

The key findings of this retrospective analysis of real-world data are that in recent times treatment for cGVHD, and in particular moderate–severe disease, continued to have an unmet need and patients continued to spend significant time in healthcare, consume specialised care resources and do not contribute to economic productivity due to sickness absence. This has both economic impacts for society, and quality of life implications for patients. Furthermore, understanding resource usage and overall costs in the management of cGVHD has implications for policy-makers, healthcare providers and industry to drive innovation and introduction of novel diagnostics and treatments to manage cGVHD.

Our present study showed that the cumulative direct medical cost of specialised healthcare during the first 3 years of follow-up in patients with moderate–severe cGVHD was approximately four- and threefold higher versus patients with non-cGVHD or mild cGVHD, respectively. Similarly, a Tunisian pharmacoeconomic study showed that the expenditure on patients with cGVHD was threefold higher versus patients with non-cGVHD [24]. A study assessing the base-case estimated costs for cord-blood transplantations reported that the presence of cGVHD was associated with direct costs of USD 2716 (EUR 2288 [adjusted for inflation for 2018]) [23]. Another study showed that, following umbilical cord transplantation, the presence of cGVHD required a mean of 12 additional days of hospitalization. This led to incremental costs of EUR 9180 (EUR 9322 [adjusted for inflation for 2018]) in the first year [22]. Our study also showed that generally patients with moderate-severe cGVHD needed more inpatient care than outpatient care than other patient severity groups, whereas in patients with non-cGVHD, the reverse trend was mostly seen. Indeed, a recent US study also described inpatient costs as the most significant cost burden in treating cGVHD patients in the first two years after HSCT [25]. Furthermore, our cost estimation for the period after 182 days post-transplant may be considered conservative given it did not the costs for HSCT or those incurred in the first 182 days post-HSCT. Within our observation period, costs did not include primary care visits or pharmacy dispensed medication.

We have shown that healthcare utilisation and sickness absence were considerably higher in patients with moderate–severe cGVHD than patients with non- and mild cGVHD. In a different analysis of our dataset, we observed greater morbidity in patients with moderate–severe cGVHD compared with non- and mild cGVHD [19]. During the third year, mean indirect cost per patient for those with non-cGVHD was EUR 1968 (median: EUR 0 [IQR: EUR 0–0]), due to skewed data. We have observed a small proportion of patients with high costs as shown by the IQR and mean cost, whereas the majority of non-cGVHD patients have shown to be economically active, thus incurring no or very low indirect costs. Nevertheless, our current analysis also revealed that the indirect cost (cumulative productivity loss) during the first 3 years of follow-up in patients with moderate–severe cGVHD was approximately twofold higher versus both patients with non-cGVHD or with mild cGVHD, respectively. High costs associated with moderate–severe cGVHD lasted for several years compared with patients with non- and mild cGVHD.

Our present analysis showed that the combined direct medical and indirect productivity costs during the first 3 years of follow-up was approximately three- and twofold higher in patients with moderate–severe cGVHD versus those with non- and mild cGVHD, respectively—posing a considerable burden on the healthcare system and to society in Sweden. In our study, 89% of patients with mild and moderate-severe cGVHD had received an income, showing the high proportion of patients working with this condition. Moreover, a longitudinal study in the United States showed that, among patients who had undergone HSCT, only 41% of patients with cGVHD had returned to work by the third year of follow-up compared with 95% of patients without cGVHD [16]. Similarly, a sensitivity analysis in the United States on the loss of wages and productivity in patients with all cases of cGVHD showed that only 37.5% of these patients would ever return to work [28]. Of those studied, 75% of patients would be expected to fully recover and only lose 3 years of earnings, the remaining 25% would each lose 20 years of earnings because of permanent disability [28]. Again, showing similar findings on the impact of work, a recent questionnaire study by the North American cGVHD Consortium (Canada and United States) assessed the insurance, employment, and financial challenges endured by patients with cGVHD who had undergone HSCT [29]. The study showed that, of respondents who reported a financial burden, 40% included ‘inability to return to work’ as a cause [29]. Further causes included the need for multiple medications/treatments (41%), frequent physician visits (32%), and losing/changing insurance (8%) [29].

In the context of costs incurred by haematological malignancies, the economic burden of chronic lymphocytic leukaemia (CLL) was assessed from a German database of 4198 patients with CLL [37]. The total mean cost incurred by a patient with CLL in Germany was revealed to be EUR 9753 per year [37]. Compared with our analysis, a twofold higher mean cost was seen for a patient with moderate–severe cGVHD during the third year (EUR 21,156 per year). Comparing our findings with other complications experienced following HSCT, a retrospective database study assessed the cost of patients with severe sinusoidal obstruction syndrome (SOS)/veno-occlusive disease and multi-organ dysfunction (MOD), a life-threatening complication associated with HSCT [38]. Of those patients assessed, 5.4% had SOS and 46% had MOD [38]. There was a 1.4-fold higher mean hospital cost in patients with SOS/MOD versus those without SOS/MOD. In relation to our findings, patients with moderate–severe cGVHD incurred threefold higher total cumulative costs during the first 3 years of follow-up versus patients with non-cGVHD. An important consideration is that our analysis does not take into account the costs for HSCT or costs incurred in the first 182 days post-HSCT. HSCT costs in patients with malignancies from 2012 have been reported as averaging EUR 141,493 in the first year post-HSCT [39].

Despite improved survival following HSCT in patients with cGVHD [12], there is high long-term sickness absence in these patients that persists for at least 10 years. An incidence model suggested that, with the steady growth in HSCTs performed in Europe, the number of patients with cGVHD will increase by approximately 7.5% in the next 5 years [7]. Therefore, there remains an unmet need for new therapies for cGVHD, and new strategies that improve disease control with fewer associated morbidities. Future analysis of cGVHD-driven expenditure may necessitate the extrapolation of these costs in the future to determine fully the projected impact this condition has on spending and social security systems. Addressing areas of cost reduction, a retrospective analysis in patients continuing secondary systemic treatments (mTOR inhibitors, ECP, and imatinib) for cGVHD for a median of 15 months indicated a potential cost benefit of continuing treatment [40]. The study showed a reduction in hospitalization costs in patients continuing secondary systemic treatments compared with those who discontinue treatment.

Our methodology focuses on the timing and extent to which patients received systemic immunomodulatory cGVHD treatment to identify those with cGVHD [19]. Moderate–severe cGVHD patients were classified based on treatment in clinical practice; defined by several potential treatment regimens encompassing co-administration of systemic corticosteroids and immunosuppressives, and ECP (Supplementary Fig. 2 in Online Resource). While treatment-specific analysis was beyond the scope of the study, systemic corticosteroids and immunosuppressant co-administration was the most common regimen for moderate–severe cGVHD patients, and approximately 10% received ECP as part of their treatment (Supplementary Fig. 2 in Online Resource).

By assessing long-term survivors (patients from 182 days post-HSCT), we have observed a high cGVHD incidence of 71.9%. Even when comparing all transplant patients in our study population (defined as 18–75 years of age with a hematological malignancy diagnosis who underwent HSCT 2006–2015), the incidence rate of cGVHD was 62%. And while this is on the higher side of the incidence rates reported by other studies using NIH criteria (30–70%; [3–5]), the lack of uptake of NIH criteria has been reported to be high as approximately 60% [18]. As we included all patients who actually received a treatment that meets the clinical indication of cGVHD, this study adds to the important discussion about underdiagnosis of cGVHD.

The limitations of our study include those inherent in real-world evidence studies. This analysis is limited by the data derived from the national registers. As sickness absence days are provided as aggregated days for the whole calendar year in the LISA Register, this may underestimate the absence rates. Additionally, there is considerable between-patient variability in costs, indicated by the IQR. However, this might be expected given the considerable heterogeneity in the presentation of the disease between different patients. Further, our criteria may not capture all patients with mild disease, specifically those with mild cGVHD limited to a single organ and those managed only with topical treatments [17]. Our retrospective definition of disease severity does not follow NIH guidelines; the NIH 2014 criteria are the standard for implementing decisions on cGVHD treatment and enrolment in clinical trials [17]. Furthermore, although the 182-day post-HSCT study period was chosen to best capture the period of cGVHD, there is a risk that some late acute GVHD patients may be included in our cGVHD classification. DRG-based hospital costing accounts for complete care needs for specialised healthcare (inpatient and outpatient); however, it does not include primary doctor visits and pharmacy collected medicine, and therefore may underestimate total cost of care.

Nonetheless, the strengths of our study include its population-based innovative design, providing real-world estimates of direct medical and indirect costs associated with cGVHD. Supportive of our methodology, register data have been used to assess morbidities in cGVHD previously in a Japanese study [41]. Sweden is an international leader in the establishment of such disease registers [36] and have been used as the data source when defining disease severity in observational studies in disease areas such as psoriasis [42] and depression [43]. Highlighting the clinical relevance of such register-based analysis, a validation study using psoriasis register data demonstrated a high degree of sensitivity and positive predictive value, within the range of accepted values used for operational research [44].

## Conclusion

In conclusion, our study based on the innovative method of defining cGVHD severity showed that cGVHD poses a high economic burden to healthcare systems and society in Sweden—in terms of both direct specialised healthcare and indirect costs—leading to a considerable economic impact. Severity of cGVHD is a major driver of these rising costs, with moderate–severe cGVHD patients spending more time in healthcare, consuming more healthcare resources and having greater productivity loss due to significant sickness absence. This indicates the importance of continued diagnostic and treatment innovation to reduce moderate–severe cGVHD, not only to maintain the clinical graft versus tumour benefits of HSCT, but also to reduce the considerable economic cost to society and patients’ quality of life.

## Electronic supplementary material

Below is the link to the electronic supplementary material.

Supplementary file1 (DOCX 52 KB)

Supplementary file2 (PDF 674 KB)
