# Supplementary material for: Real-world study of direct medical and indirect costs and time spent in healthcare in patients with chronic graft versus host disease
Source: Eur J Health Econ. 2020 Dec 4;22(1):169–80. doi: 10.1007/s10198-020-01249-x (PMC7822787; doi:10.1007/s10198-020-01249-x)
Supplement: Supplementary file 1 — Supplementary file1 (DOCX 52 KB) [file 10198_2020_1249_MOESM1_ESM.docx]

**Electronic Supplementary Material: Online Resources**

**Journal:** *The European Journal of Health Economics*

**Title:** Real-world study of direct medical and indirect costs and time spent in healthcare in patients with chronic graft versus host disease

Frida Schain^1,2,3^ ● Nurgul Batyrbekova^4,5^ ● Johan Liwing^1,6^ ● Simona Baculea^7^ ● Thomas Webb^7^ ● Mats Remberger^8^ ● Jonas Mattsson^9,10,11^

^1^Janssen Global Services, Stockholm, Sweden

^2^Department of Medicine, Division of Hematology, Karolinska Institutet, Solna, Sweden

^3^Schain Research, Bromma, Sweden

^4^Department of Medical Epidemiology and Biostatistics, Karolinska Institutet, Stockholm, Sweden.

^5^Scandinavian Development Services, Stockholm, Sweden

^6^Department of Medicine, Division of Hematology, Karolinska Institutet, Huddinge, Sweden

^7^Janssen Global Services, High Wycombe, UK

^8^KFUE, Uppsala University Hospital and Institution of Medical Science, Uppsala University, Uppsala, Sweden

^9^Department of Oncology and Pathology, Karolinska Institutet, Stockholm, Sweden

^10^Princess Margaret Cancer Centre, University of Toronto, Gloria and Seymour Epstein Chair in Cell Therapy and Transplantation, Toronto, ON, Canada

^11^Department of Medicine, University of Toronto, Toronto, Canada

**Correspondence to:** Frida Schain, Karolinska Institutet, Department for Medicine, Division of Hematology, Stockholm, Sweden

**Tel:** +46 72 330 53 13

**Online Resource 1:** Supplementary Figures 1 and 2; Supplementary Tables 1-3; Supplementary Methods
