# Supplementary material for: Real-world study of direct medical and indirect costs and time spent in healthcare in patients with chronic graft versus host disease
Source: Eur J Health Econ. 2020 Dec 4;22(1):169–80. doi: 10.1007/s10198-020-01249-x (PMC7822787; doi:10.1007/s10198-020-01249-x)
Supplement: Supplementary file 2 — Supplementary file2 (PDF 674 KB) [file 10198_2020_1249_MOESM2_ESM.pdf]

Online Resource: Supplementary Appendix

Supplementary Fig. 1 Schematic of study period and design.

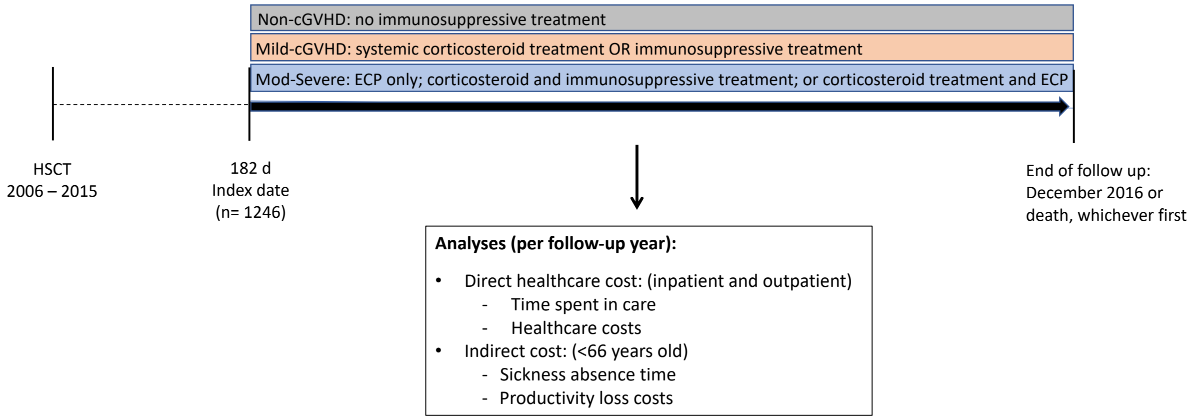

Supplementary Fig. 1 Schematic of study period and design. Patients were identified in the Patient Register who underwent HSCT and survived >182 days. This was the start of the observation period. Patients were classified as non-, mild or moderate-severe cGVHD based on treatment they received (as described in Mattsson et al. [19]), recorded in the Patient and Prescribed Drug Register. Patients were followed until December 31st or death, whichever occurred first. Analyses of Direct healthcare costs (Patient Register; inpatient and outpatient care), and indirect productivity costs (LISA; sickness-absence and salary loss in patients <66 years of age)

Supplementary Fig. 2 cGVHD Patient Classification Definition

a.

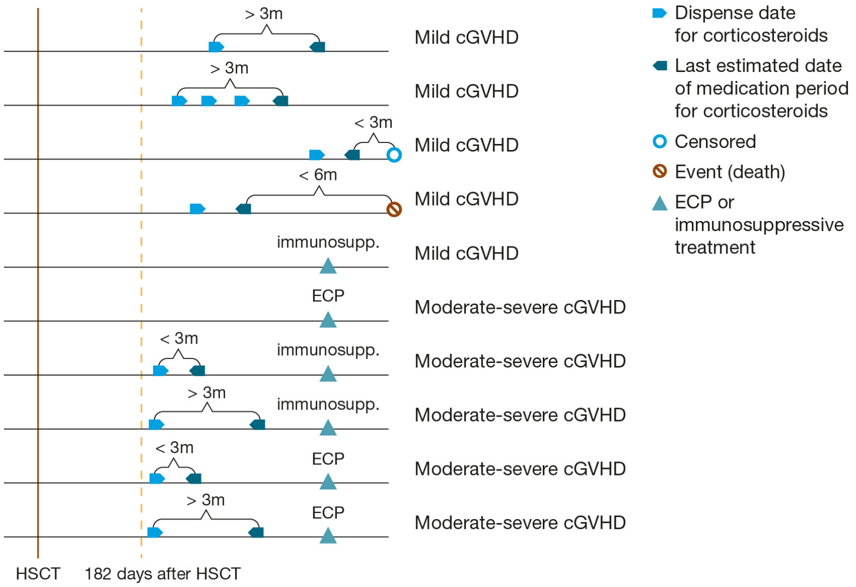

b.

cGVHD severity	Treatment type received	Patients
mild cGVHD	Systemic corticosteroids	196
mild cGVHD	Immunosuppressants	149
moderate-severe cGVHD	ECP	4
moderate-severe cGVHD	ECP, Systemic corticosteroids	11
moderate-severe cGVHD	ECP, Systemic corticosteroids, Immunosuppressants	36
moderate-severe cGVHD	ECP, Immunosuppressants	2
moderate-severe cGVHD	Systemic corticosteroids, Immunosuppressants	498
	Total	896

Supplementary Fig. 2 cGVHD Patient Classification Definition. cGVHD classification by treatment criteria in patients who survived 182 days following HSCT (a) and the number patients in each treatment regimen subcategory (b). *cGVHD* chronic graft versus host disease, *ECP* extracorporeal photopheresis, *HSCT* haematopoietic stem cell transplantation, *m* months. Supp Fig 2b was included in the conference abstract Mattsson et al. [19], and reproduced with permission as per requirements of the American Society for Blood and Marrow Transplantation and Center for International Blood and Marrow Transplant Research. Copyright Elsevier (2019).

Supplementary Table 1 Direct medical cost in patients with non-, mild, and moderate-severe cGVHD

Patients with non-cGVHD								
Follow-up year	Patient-years	Outpatient care costs (EUR)	Inpatient care costs (EUR)	Total direct medical costs (EUR)	Mean direct medical cost per patient-year (EUR)	Median direct medical cost per patient-year (EUR)	IQR 1 (EUR)	IQR 3 (EUR)
1	282	2,623,891	3,005,337	5,629,228	19,962	9463	6354	28,351
2	241	772,006	736,444	1,508,450	6259	2605	1443	5411
3	197	454,176	253,260	707,436	3591	1743	637	3028
4	166	339,837	219,343	559,180	3369	1240	404	2784
5	137	205,737	92,842	298,579	2179	808	0	1949
6	106	141,493	88,807	230,301	2173	727	0	1876
7	79	73,475	17,140	90,615	1147	374	0	1182
8	52	46,475	28,426	74,901	1440	535	0	1089
9	37	26,543	6699	33,243	898	0	0	1075
10	22	7394	0	7394	336	0	0	0

Patients with mild cGVHD patients								
Follow-up year	Patient years	Outpatient care costs (EUR)	Inpatient care costs (EUR)	Total direct medical costs (EUR)	Mean direct medical cost per patient-year (EUR)	Median direct medical cost per patient-year (EUR)	IQR 1 (EUR)	IQR 3 (EUR)
1	309	3,414,271	4,035,612	7,449,883	24,110	13,433	8576	31,418
2	260	1,152,059	1,703,975	2,856,034	10,985	3957	1822	9380
3	210	693,201	972,188	1,665,390	7930	1875	743	5020
4	160	353,570	293,992	647,562	4047	1457	368	3072
5	130	409,856	409,826	819,681	6305	1336	369	3328
6	103	246,584	248,174	494,758	4803	771	0	2287
7	77	125,233	82,791	208,025	2702	559	0	1550
8	58	65,596	65,929	131,525	2268	676	0	1406
9	40	41,632	130,954	172,586	4315	0	0	781
10	22	22,171	71,029	93,200	4236	218	0	1027

Patients with moderate-severe cGVHD								
Follow-up year	Patient years	Outpatient care costs (EUR)	Inpatient care costs (EUR)	Total direct medical costs (EUR)	Mean direct medical cost per patient-year (EUR)	Median direct medical cost per patient-year (EUR)	IQR 1 (EUR)	IQR 3 (EUR)
1	519	7,927,899	8,996,027	16,923,926	32,609	20,933	11,620	44,230
2	449	3,622,148	4,735,843	8,357,991	18,615	8636	3284	22,838
3	374	2,407,401	2,954,154	5,361,555	14,336	4979	1388	15,408
4	303	1,425,586	1,835,426	3,261,012	10,762	2763	725	10,220
5	236	1,003,161	1,221,782	2,224,942	9428	2104	407	10,344
6	186	637,789	836,575	1,474,363	7927	1519	430	6308
7	148	456,416	502,272	958,688	6478	1140	0	5055
8	104	204,729	225,951	430,680	4141	398	0	2682
9	64	141,410	138,554	279,964	4374	565	0	2951
10	38	46,816	55,401	102,217	2690	0	0	1163

cGVHD chronic graft versus host disease, *IQR* interquartile range

Supplementary Table 2 Indirect costs (productivity loss due to sickness absence) in patients with non-, mild and moderate-severe cGVHD

Patients with non-cGVHD						
Follow-up year	Patient-years	Total PL (EUR)	Mean PL per patient-year (EUR)	Median PL per patient-year (EUR)	IQR 1 (EUR)	IQR 3 (EUR)
1	243	5,622,556	23,138	24,252	6056	37,518
2	188	1,116,836	5941	0	0	8732
3	154	303,093	1968	0	0	0
4	126	152,445	1210	0	0	0
5	91	77,386	850	0	0	0
6	71	47,580	670	0	0	0
7	53	23,566	445	0	0	0
8	38	23,494	618	0	0	0
9	22	23,893	1086	0	0	0
10	12	1058	88	0	0	0

Patients with mild cGVHD						
Follow-up year	Patient-years	Total PL (EUR)	Mean PL per patient-year (EUR)	Median PL per patient-year (EUR)	IQR 1 (EUR)	IQR 3 (EUR)
1	272	5,864,619	21,561	23,283	134	35,240
2	212	1,909,641	9008	0	0	14,456
3	165	798,488	4839	0	0	4952
4	125	456,332	3651	0	0	2975
5	100	503,996	5040	0	0	2975
6	81	337,318	4164	0	0	2308
7	55	199,570	3629	0	0	2739
8	43	47,393	1102	0	0	0
9	27	40,238	1490	0	0	0
10	19	26,574	1399	0	0	0
Patients with moderate-severe cGVHD						
Follow-up year	Patient years	Total PL (EUR)	Mean PL per patient-year (EUR)	Median PL per patient-year (EUR)	IQR 1 (EUR)	IQR 3 (EUR)
1	465	10,196,611	21,928	23,893	0	35,921
2	376	4,401,457	11,706	4355	0	22,897
3	296	2,570,296	8683	0	0	16,032
4	229	1,283,839	5606	0	0	8299
5	170	759,471	4467	0	0	4275
6	138	531,673	3853	0	0	0
7	102	481,599	4722	0	0	2590
8	64	193,740	3027	0	0	0
9	38	92,348	2430	0	0	0
10	20	13,331	667	0	0	0

cGVHD chronic graft versus host disease, *IQR* interquartile range, *PL* productivity loss

Supplementary Table 3 Direct medical and indirect costs in patients with non-, mild and moderate-severe cGVHD

Patients with non-cGVHD						
Follow-up year	Patient years	Total cost (EUR)	Mean cost per patient-year (EUR)	Median cost per patient-year (EUR)	IQR 1 (EUR)	IQR 3 (EUR)
1	282	11,251,784	39,888	37,540	15,779	61,910
2	241	2,625,286	10,864	3607	1605	12,771
3	197	1,010,529	5107	1774	637	3749
4	166	711,626	4267	1243	404	2918
5	137	375,965	2732	940	0	2216
6	106	277,880	2603	735	0	2043
7	79	114,181	1430	374	0	1228
8	52	98,394	1889	559	0	1188
9	37	57,136	1539	0	0	1469
10	22	8452	368	0	0	534

Patients with mild cGVHD						
Follow-up year	Patient years	Total cost (EUR)	Mean cost per patient-year (EUR)	Median cost per patient-year (EUR)	IQR 1 (EUR)	IQR 3 (EUR)
1	309	13,314,502	42,974	37,148	17,057	65,940
2	260	4,765,676	18,327	6706	2261	27,187
3	210	2,463,878	11,699	2354	773	11,494
4	160	1,103,895	6875	1521	368	6767
5	130	1,323,677	10,179	1541	369	5247
6	103	832,076	8039	928	110	4344
7	77	407,595	5263	661	0	2954
8	58	178,918	3042	681	0	1571
9	40	212,824	5291	0	0	1608
10	22	119,774	5277	302	0	3648
Patients with moderate-severe cGVHD						
Follow-up year	Patient years	Total cost (EUR)	Mean cost per patient-year (EUR)	Median cost per patient-year (EUR)	IQR 1 (EUR)	IQR 3 (EUR)
1	519	27,120,537	52,209	45,176	25,981	70,862
2	449	12,759,448	28,413	17,444	5187	42,793
3	374	7,931,851	21,156	8830	1718	27,431
4	303	4,544,851	14,999	3333	803	18,595
5	236	2,984,414	12,616	2525	430	14,905
6	186	2,006,036	10,752	1695	430	9617
7	148	1,440,287	9687	1337	0	8519
8	104	624,421	5984	399	0	4300
9	64	372,312	5756	565	0	3176
10	38	115,548	2997	0	0	2239

cGVHD chronic graft versus host disease, *IQR* interquartile range

Supplementary Methods

Sickness absence

The sickness absence rate for the follow-up year was then defined as the total number of sickness absence days divided by the total contributed follow-up time. As the follow-up years were not aligned with calendar years specified in the Longitudinal Integration Database for Health Insurance and Labour Market Studies (LISA) Register, we split aggregated yearly sickness-absence days in LISA according to the follow-up years in our cohort data. We censored the remaining part of observation time if it occurred after 31 December 2016. Also, we censored patients after the tenth follow-up year from the 182 days post-haematopoietic stem cell transplantation start date. If a patient reached the age of > 65 years at the follow-up year, then we assumed that this patient was receiving a pension and we censored the remaining time for this patient. For patients who had missing final years in LISA due to their short follow-up time (< 365 days), we implemented the method of last observation carried forward. If a patient did not have any records in LISA, then this patient was excluded from the analysis of sickness absence.

Healthcare utilisation

Incidence rate ratios of healthcare resource use for the follow-up year was calculated as the total number of days in inpatient and outpatient care divided by the total contributed follow-up time for that follow-up year.

Mathematically, for a given follow-up year where j = 1,…,10:

$$\text{Healthcare utilisation rate}_j = \frac{\sum_{i=1}^{N_j} (\text{time in care})_{ij}}{\sum_{i=1}^{N_j} (\text{patient-time at risk})_{ij}} * 100$$

where i = 1,….,N_j is the number of patients in the jth follow-up year.
